# Flea-Associated Bacterial Communities across an Environmental Transect in a Plague-Endemic Region of Uganda

**DOI:** 10.1371/journal.pone.0141057

**Published:** 2015-10-20

**Authors:** Ryan Thomas Jones, Jeff Borchert, Rebecca Eisen, Katherine MacMillan, Karen Boegler, Kenneth L. Gage

**Affiliations:** 1 Department of Microbiology and Immunology, Montana State University, Bozeman, Montana, United States of America; 2 Montana Institute on Ecosystems, Montana State University, Bozeman, Montana, United States of America; 3 Division of Vector-Borne Disease; Centers for Disease Control and Prevention, Fort Collins, Colorado, United States of America; University of Helsinki, FINLAND

## Abstract

The vast majority of human plague cases currently occur in sub-Saharan Africa. The primary route of transmission of *Yersinia pestis*, the causative agent of plague, is via flea bites. Non-pathogenic flea-associated bacteria may interact with *Y*. *pestis* within fleas and it is important to understand what factors govern flea-associated bacterial assemblages. Six species of fleas were collected from nine rodent species from ten Ugandan villages between October 2010 and March 2011. A total of 660,345 16S rRNA gene DNA sequences were used to characterize bacterial communities of 332 individual fleas. The DNA sequences were binned into 421 Operational Taxonomic Units (OTUs) based on 97% sequence similarity. We used beta diversity metrics to assess the effects of flea species, flea sex, rodent host species, site (i.e. village), collection date, elevation, mean annual precipitation, average monthly precipitation, and average monthly temperature on bacterial community structure. Flea species had the greatest effect on bacterial community structure with each flea species harboring unique bacterial lineages. The site (i.e. village), rodent host, flea sex, elevation, precipitation, and temperature also significantly affected bacterial community composition. Some bacterial lineages were widespread among flea species (e.g. *Bartonella* spp. and *Wolbachia* spp.), but each flea species also harbored unique bacterial lineages. Some of these lineages are not closely related to known bacterial diversity and likely represent newly discovered lineages of insect symbionts. Our finding that flea species has the greatest effect on bacterial community composition may help future investigations between *Yersinia pestis* and non-pathogenic flea-associated bacteria. Characterizing bacterial communities of fleas during a plague epizootic event in the future would be helpful.

## Introduction

Since 2000, greater than 95% of reported cases of human plague have occurred in sub-Saharan Africa [1]. Models incorporating 10-year meteorological data and human plague incidence data found that, in Uganda, plague risk increases in sites located above 1,300 meters in elevation and with increased (but not continuous) rains in February, October, and November [2]. In addition, the number of human plague cases in the West Nile region was negatively associated with dry season rainfall and positively with rainfall during the interval rainy season that immediately precedes plague transmission season [3]. Increased rainfall may increase primary production, which may, in turn, increase rodent and flea abundance [2,4,5]. Increases in flea abundance are predicted to increase the risk of a plague epizootic event [6].

In addition to flea abundance, flea-associated microbial communities may also contribute to plague transmission. *Yersinia pestis*, the causative agent of plague, reduces the abundance of or completely eliminates specific bacterial lineages within fleas [7], and exposing laboratory-reared fleas to diverse wild-type microbial communities increases transmission of *Y*. *pestis* [8]. Although determining the presence of particular lineages (e.g. *Rickettsia* spp., *Bartonella* spp., *Yersinia pestis*) in wild fleas has been routine, the characterization of entire bacterial communities of wild fleas has been limited [9,10]. The microbial compositions of two closely related fleas (*Oropsylla hirsuta* vs. *Oropsylla tuberculata cynomuris*) and of two more distantly related fleas (*Orchopeas leucopus* vs. *Ctenophthalmus pseudagyrtes*) were not found to differ [9,10]. However, flea-associated bacterial communities shifted drastically over three years in both species of flea studied [10]. Shifts in microbial communities over time are often due to concomitant shifts in environmental conditions [11–15], but environmental conditions have not previously been explored in relation to insect-associated bacterial communities.

Symbionts of disease vectors may mediate the spread of disease through negative or positive interactions with pathogens. *Wolbachia*-positive mosquitos have suppressed rates of infection by dengue virus, Chikungunya virus, West Nile virus, and *Plasmodium* spp. [16–18], and introducing *Wolbachia*-positive mosquitos to a natural population has proven to be an effective means to decrease the number of potential vectors of human disease [19]. The entire insect-associated microbiome can also influence pathogen persistence; dengue virus titers in sterile *Aedes aegypti* midguts are significantly higher than titers in *A*. *aegypti* with wildtype microbiomes [20]. These negative effects of vector-associated microbes on pathogens do not seem to occur between flea-associated bacteria and *Y*. *pestis*: exposing ‘germ-free’ fleas to wildtype microbes increases transmission of *Y*. *pestis* [8] and infecting wild fleas (with wild-type microbiomes) with *Y*. *pestis* eliminates specific bacterial lineages within fleas [7]. Flea-borne viruses have yet to be studied, but interactions between viruses and *Y*. *pestis* may also alter the ability of fleas to transmit *Y*. *pestis* and would be a novel research pursuit in the future.

In this study, we characterized the bacterial communities of six flea species collected from nine species of rodents in March 2011 from ten sites in a plague-endemic area of Uganda. For the two most abundant flea species, we analyzed additional samples collected in October and December 2010. Due to our sampling strategy, we can test for the effects of rodent host, flea species, site, environmental conditions, and time on flea-associated bacterial communities.

## Materials and Methods

### Flea Samples

Field collection permits are not required in Uganda, but all protocols for this work were reviewed and approved by the Uganda Virus Research Institute Science and Ethics Committee, the Uganda National Council of Science and Technology, and the Uganda President’s Office. All research protocols involving animals (e.g. trapping animals to capture fleas) was also approved by the Animal Care and Use Committee of the Division of Vector-Borne Disease at the United States Centers for Disease Control and Prevention. Fleas were collected at three times across ten sites in Uganda [21]. Briefly, Tomahawk and Sherman live traps were used, traps were set at dusk and and retrieved the next morning, animals were released after sampling, and no animals died during trapping; for full details, please see the original publication that details flea and rodent diversity across these sites [21]. The three collection times correspond to the peak of the primary rainy season (October 2010), the dry season (November/December 2010), and the beginning of the secondary rainy season (March 2011). We used a subset of these fleas to investigate flea-associated bacterial communities. *Xenopsylla brasiliensis* and *Xenopsylla cheopis* were examined from the three different collection periods; *Ctenophthalmus calceatus cabirus*, *Dinopsyllus lypusus*, *Stivalius torvus*, and *Xenopsylla nubica* were examined from the March 2011 collection (Table 1). Fleas analyzed in this study were collected from nine taxa of rodents: *Aethomys hindei*, *Arvicanthis niloticus*, *Crocidura* spp., *Lophuromys flavopunctatus*, *Lemniscomys striatus*, *Mastomys* spp., *Rattus rattus*, *Taterillus emini*, and *Tatera valida* (Table 2).

**Table 1:** Number of individual fleas analyzed from each flea species across three collection periods.

Site	Ccc	Dl	St	Xb			Xc			Xn
	Mar	Mar	Mar	Oct	Nov/Dec	Mar	Oct	Nov/Dec	Ma	Mar
1	-	-	-	-	-	-	2	4	7	-
2	-	-	-	-	-	-	9	15	16	-
3	-	-	-	-	-	-	13	10	14	-
4	-	-	-	-	-	-	11	6	13	-
5	-	-	-	-	-	-	8	8	17	7
6	-	-	-	-	-	-	3	7	14	9
7	-	-	-	-	-	4	-	-	1	-
8	-	-	-	9	7	12	-	-	-	-
9	13	12	16	9	10	14	-	-	-	-
10	-	4	-	2	9	16	-	-	-	-
Total	13	16	16	20	26	46	46	50	82	16

Ccc: *Ctenophthalmus calceatus cabirus*, Dl: *Dinopsyllus lypusus*, St: *Stivalius torvus*, Xb: *Xenopsylla brasiliensis*, Xc: *Xenopsylla cheopis*, Xn: *Xenopsylla nubica*.

Oct: October, 2010; Nov/Dec: November/December, 2010; Mar: March, 2011.

**Table 2:** Distribution of fleas across mammalian hosts.

Host	Ccc	Dl	St	Xb	Xc	Xn
*A*. *hindei*					8	
*A*. *niloticus*	9	12		37	72	
*Crocidura* spp.		1	15	3	8	
*L*. *flavopunctatus*			1			
*L*. *striatus*		3				
*Mastomys* spp.	4			8	15	
*R*. *rattus*				44	75	
*T*. *emini*						2
*T*. *valida*						14
Total	13	16	16	92	178	16

Fleas: Ccc: *Ctenophthalmus calceatus cabirus*, Dl: *Dinopsyllus lypusus*, St: *Stivalius torvus*, Xb: *Xenopsylla brasiliensis*, Xc: *Xenopsylla cheopis*, Xn: *Xenopsylla nubica*.

Hosts: *A*. *hindei*: *Aethomys hindei; A*. *niloticus*: *Arvicanthis niloticus; Crocidura* spp.: *Crocidura* species; *L*. *flavopunctatus*: *Lophuromys flavopunctatus; L*. *striatus*: *Lemniscomys striatus; M*. *natalensis*: *Mastomys* species*; R*. *rattus*: *Rattus rattus; T*. *emini*: *Taterillus emini; T*. *valida*: *Tatera valida*.

*DNA Extractions*.

Fleas were stored in 70% ethanol upon collection. Prior to DNA extraction, individual fleas were surface-sterilized by soaking in 10% bleach for 30 seconds and then washed twice with 100% ethanol. Surface-sterilized fleas were subjected to 20 minutes of mechanical lysis using a Retsch MM301 homogenizer, and then DNA was extracted using the MO BIO PowerSoil-htp 96 Well Soil DNA Isolation Kit (Carlsbad, CA) with the standard protocol.

### DNA Sequencing

We amplified the V1 and V2 hypervariable regions of the 16S rRNA gene using previously described primers: the forward primer (5’-GCCTTGCCAGCCCGCTCAGTCAGAGTTTGATCCTGGCTCAG-3’) contains the 16S rRNA gene 27f primer, the 454 Life Sciences primer B sequence, and a two-base ‘TC’ linker; the reverse primer (5′-GCCTCCCTCGCGCCATCAGNNNNNNNNNNNNCATGC TGCCTCCCGTAGGAGT-3′) contains a 12 bp error-correcting barcode, the 16S rRNA gene 338r primer, the Life Sciences primer A sequence, and a two-base ‘CA’ linker [22]. We amplified the DNA samples using the following conditions: Initial denaturation at 94°C for 5 min; then 35 cycles of 94°C for 45s, 50°C for 30s, 72°C for 90s; with a final extension at 72°C for 10 min. Each amplification was performed in triplicate and PCR products from the three independent reactions were combined and cleaned using the MO BIO UltraClean-htp 96 Well PCR Clean-Up Kit (Carlsbad, CA). The concentration of each sample was estimated using the Quant-iT PicoGreen dsDNA Assay Kit (Life Technologies, Carlsbad, CA). Normalized and cleaned bar-tagged PCR products were combined into a single sample and sent to EnGenCore (Columbia, SC) for DNA sequencing on a Roche Genome Sequencer FLX using Titanium reagents.

### Sequence Analysis

We analyzed DNA sequence data using QIIME v1.8 [23]. Sequences were assigned to their flea sample based on unique barcodes and were filtered using QIIME’s default quality settings. Sequences were truncated to 280 basepairs and Operational Taxonomic Units (OTUs) were selected using the uclust algorithm and a 97% sequence similarity threshold [24]. The most abundant sequence within an OTU was chosen as its representative sequence, and representative sequences were aligned using PyNAST [25]. Aligned sequences were filtered against the greengenes core set alignment and screened for chimeras using ChimeraSlayer and chimeric sequences were removed from the dataset. DNA sequences representing less than 0.005% of all sequences were removed from the dataset. Flea samples with less than 300 DNA sequences were removed from the dataset. The final dataset included 660,345 DNA sequences from 332 fleas (range: 305–4279 DNA sequences per flea). These DNA sequences were binned into 421 OTUs (Accession #’s: KT589425 –KT589833). We assigned taxonomic classifications to the OTUs based on the RDP database, as implemented within QIIME. We estimated a phylogeny of the OTUs using FastTree [26].

### Alpha Diversity

Alpha diversity is a measure of diversity at a local scale [27]; here it refers to the amount of bacterial diversity found within an individual flea. We used flea samples represented by at least 1,000 DNA sequences (n = 282) to estimate alpha diversity. We rarefied the dataset to 1,000 (i.e. 1000 DNA sequences were randomly chosen from each flea sample). From this rarefied dataset, alpha diversity was estimated in two ways using QIIME v1.8: observed species and phylodiversity. Observed species is simply the number of unique OTUs represented in each sample. Phylodiversity is a phylogenetic alpha diversity metric and represents the sum of branch lengths represented by a single community given a phylogenetic tree constructed using all potential community members [28].

### Beta Diversity

For beta diversity measurements, we rarefied the dataset to 300 (i.e. 300 DNA sequences were randomly chosen from each flea sample). Pairwise dissimilarity matrices were created in three ways using QIIME v1.8: Bray-Curtis, UniFrac, and Weighted UniFrac [29–31]. These metrics differ in how they assess community membership: the Bray-Curtis distance uses bacterial OTU presence and abundance to compare communities, but does not account for phylogenetic relatedness of the OTUs; UniFrac is a measure of shared phylogenetic diversity, as assessed by shared branch lengths between the communities; Weighted UniFrac is similar to UniFrac but also accounts for the relative abundance of OTUs. Each of these metrics provides dissimilarity values between individual communities with the value ranging from 0 (exact same communities in both samples) to 1 (no overlap in community membership). The effects of flea species, site, host, and elevation on bacterial community composition across all samples were tested using an Analysis of Similarity as implemented in QIIME v1.8. Likewise, we used an Analysis of Similarity to test the effects of host, site, collection date, and flea sex on bacterial community composition within flea species. We also tested the effects of environmental conditions on bacterial community composition. We used Euclidean distances to create pairwise dissimilarity matrices of elevation, mean annual precipitation, average monthly precipitation, and average monthly temperature for each sample. Each pairwise dissimilarity matrix for each environmental variable was compared individually to the bacterial community dissimilarity matrices using a Mantel test implemented in QIIME v1.8. Environmental conditions were previously described [21]. Finally, principal coordinates of the bacterial community dissimilarity matrices were created and used to generate 2-dimensional plots based on flea species.

## Results

**Fig 1:**
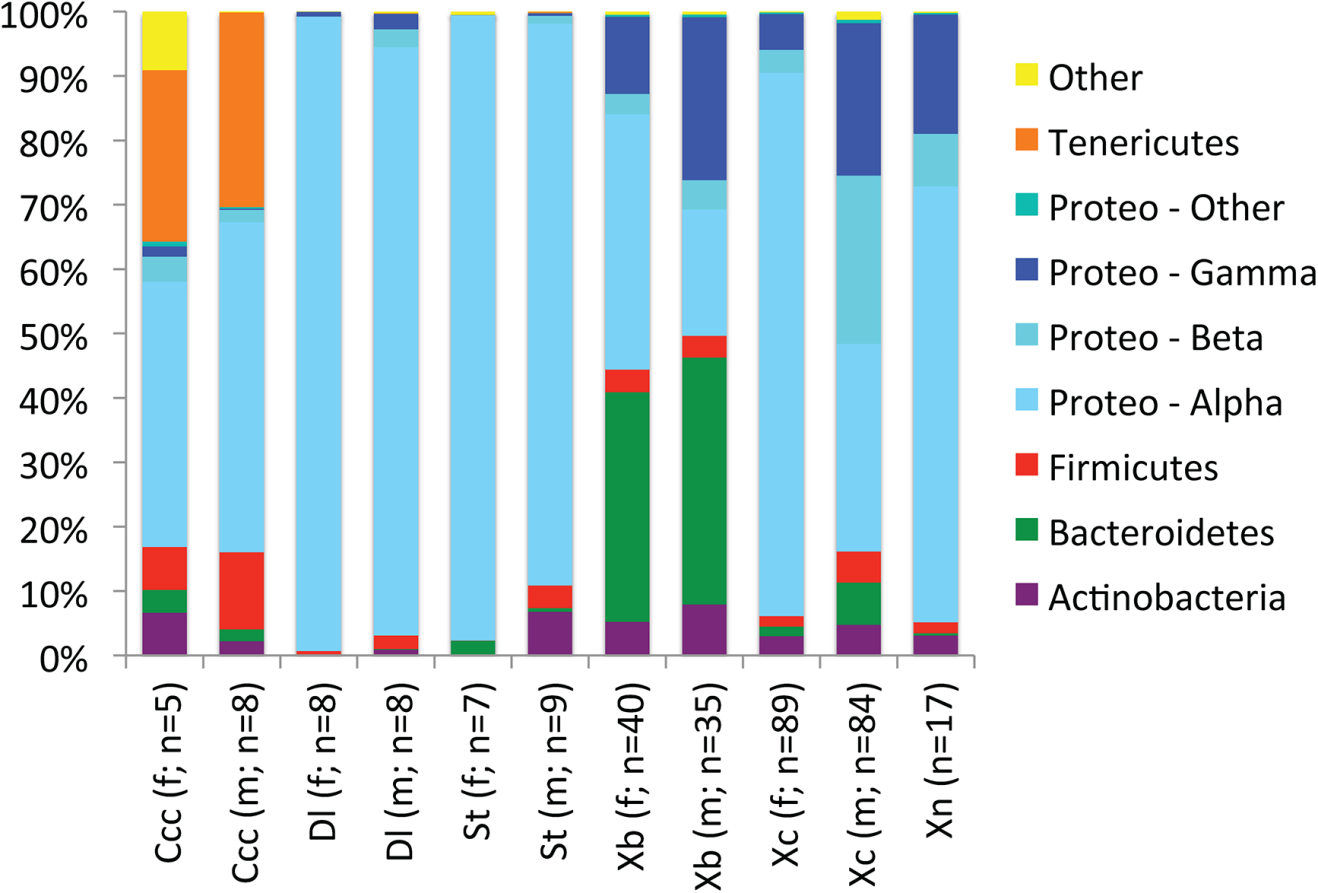
Average relative abundances of bacterial phyla within flea species. Proteobacteria were further divided based on Class: Alphaproteobacteria, Betaproteobacteria, and Gammaproteobacteria. Ccc: *Ctenophthalmus calceatus cabirus*, Dl: *Dinopsyllus lypusus*, St: *Stivalius torvus*, Xb: *Xenopsylla brasiliensis*, Xc: *Xenopsylla cheopis*, Xn: *Xenopsylla nubica*. *Xenopsylla nubica* were not sexed.

A total of 660,345 DNA sequences were grouped into 421 OTUs based on 97% sequence similarity. The vast majority of bacteria within the six flea species belonged to four bacterial phyla: Actinobacteria, Bacteroidetes, Firmicutes, and Proteobacteria (Fig 1). Within *C*. *calceatus cabirus*, a *Spiroplasma* species was also common (Table 3).

**Table 3:** Average relative abundances of most common bacterial OTUs detected in Ugandan fleas.

Taxonomic Classification	BLAST	Ccc (f)	Ccc (m)	Dl (f)	Dl (m)	St (f)	St (m)	Xb (f)	Xb (m)	Xc (f)	Xc (m)	Xn
(# individuals)	%	(5)	(8)	(8)	(8)	(7)	(9)	(40)	(35)	(89)	(84)	(16)
*Bartonella sp*.	99	7.4%	23.8%	35.6%	19.7%	8.3%	58.7%	1.2%	1.0%	3.0%	3.6%	49.5%
*Wolbachia sp*.	98	1.7%	1.1%	23.7%	13.0%	9.3%	1.1%	35.8%	5.9%	69.4%	16.1%	4.0%
*Wolbachia sp*.	98	24.8%	10.5%	0.0%	0.4%	71.4%	0.3%	0.0%	0.5%	0.0%	0.0%	0.0%
*Lariskella sp*.*	100	3.6%	4.2%	35.9%	54.0%	0.0%	1.4%	0.0%	0.0%	0.8%	0.0%	0.0%
*Cardinium sp*.	99	0.1%	0.2%	0.0%	0.0%	0.0%	0.4%	34.8%	36.8%	1.0%	0.1%	0.0%
*Spiroplasma sp*.*	99	26.3%	29.7%	0.0%	0.1%	0.0%	0.2%	0.0%	0.0%	0.0%	0.0%	0.0%
*Bartonella sp*.*	100	0.5%	7.3%	0.0%	0.0%	3.4%	22.1%	0.0%	0.0%	0.8%	0.0%	0.0%
Pasteurellaceae (family)	90	0.0%	0.0%	0.0%	0.0%	0.0%	0.0%	10.1%	21.1%	0.0%	0.0%	0.0%
Pasteurellaceae (family)	96	0.0%	0.0%	0.0%	0.0%	0.0%	0.0%	0.8%	0.5%	4.9%	21.1%	1.7%
Betaproteobacteria (class)	93	0.0%	0.0%	0.0%	0.0%	0.0%	0.0%	1.7%	0.3%	1.0%	21.3%	4.5%
*Propionibacterium acnes*	100	3.8%	0.8%	0.1%	0.5%	0.1%	0.4%	3.3%	5.0%	1.8%	3.1%	1.7%
Pasteurellaceae (family)	95	0.0%	0.0%	0.0%	0.0%	0.0%	0.0%	0.0%	0.0%	0.1%	0.2%	15.4%

Ccc: *Ctenophthalmus calceatus cabirus*, Dl: *Dinopsyllus lypusus*, St: *Stivalius torvus*, Xb: *Xenopsylla brasiliensis*, Xc: *Xenopsylla cheopis*, Xn: *Xenopsylla nubica*. Taxonomy of bacterial DNA sequences was determined using the Ribosomal Database Project classification scheme.

*: The taxonomy of DNA sequence was further classified using BLAST against GenBank’s nucleotide database.

Of the 421 OTUs detected, only 12 represented at least 1% of bacteria detected across all flea species, on average (Table 3). The most common and widespread OTU was a lineage within the *Bartonella* genus, and the next four most common lineages were those related to known endosymbionts (e.g. *Wolbachia*, *Cardinium*). Besides the most common *Bartonella* lineage and the most common *Wolbachia* lineage, most of the common lineages tended to be dominant community members within one flea species but rare community members in other flea species. Three lineages within the Pasteurellaceae were abundant in one species, but rare or absent in other species (Table 3).

**Fig 2:**
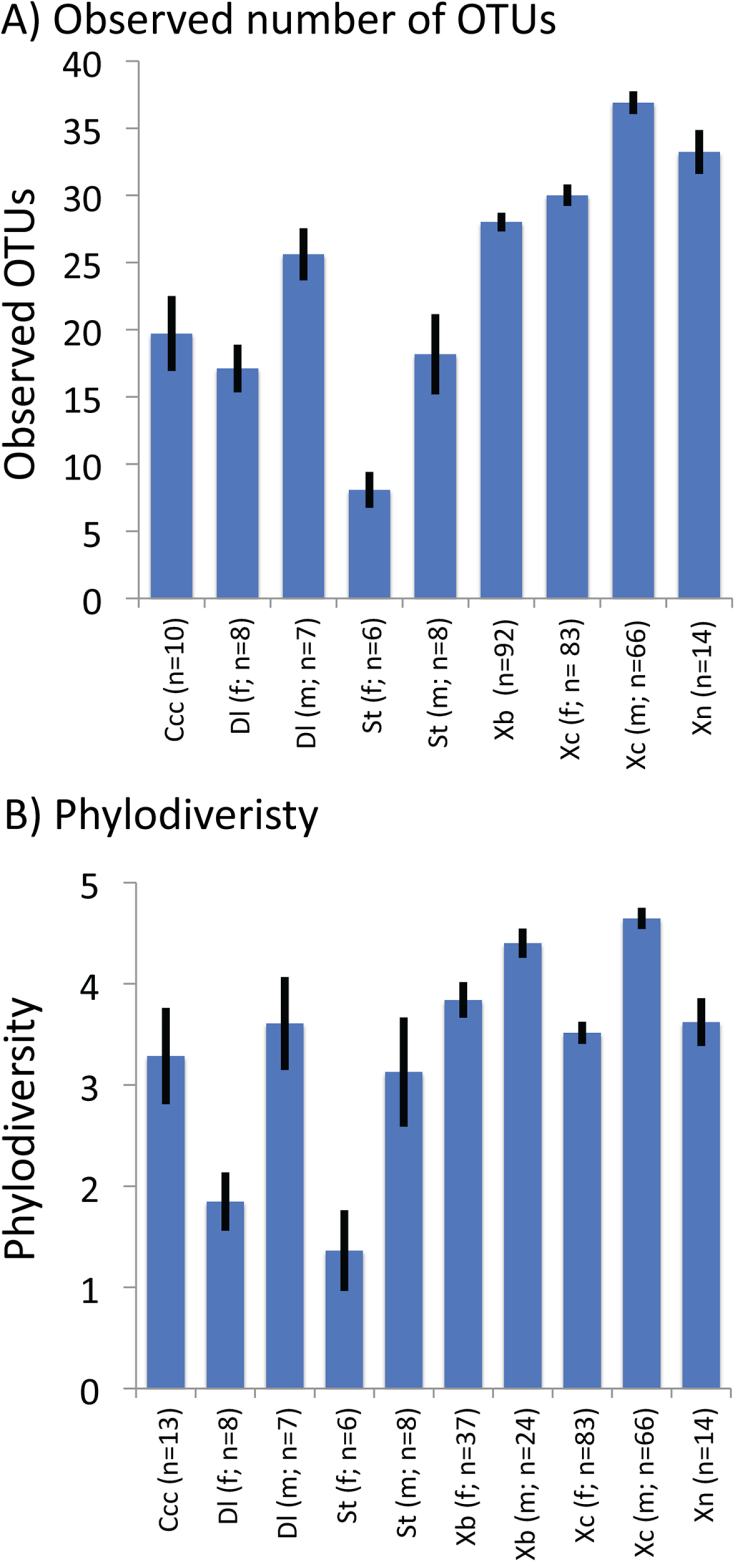
Estimates of alpha diversity for flea-associated bacterial communities. Alpha diversity was measured as the total number of observed OTUs detected in a subset of 1000 randomly chosen sequences from a sample (A) and as phylodiversity of bacteria within a sample (B). Diversity did not significantly differ between males and females of *C*. *c*. *cabirus* and sex was not determined for *X*. *nubica*. The number of observed species did not significantly differ in *X*. *brasiliensis*. In all other comparisons, male fleas harbored significantly more diversity based on student’s t-tests. Ccc: *Ctenophthalmus calceatus cabirus*, Dl: *Dinopsyllus lypusus*, St: *Stivalius torvus*, Xb: *Xenopsylla brasiliensis*, Xc: *Xenopsylla cheopis*, Xn: *Xenopsylla nubica*.

The observed number of OTUs within fleas ranged from 8.1, on average, in *S*. *torvus* females to 36.9, on average, in *X*. *cheopis* males (Fig 2A). Females had significantly less observed OTUs than males in *D*. *lypusus*, *S*. *torvus*, and *X*. *cheopis*. The phylodiversity ranged from 1.36%, on average, in female *S*. *torvus* to 4.65%, on average, in male *X*. *cheopis* (Fig 2B). Females had significantly less phylodiversity than males in *D*. *lypusus*, *S*. *torvus*, *X*. *brasiliensis*, and *X*. *cheopis*.

**Fig 3:**
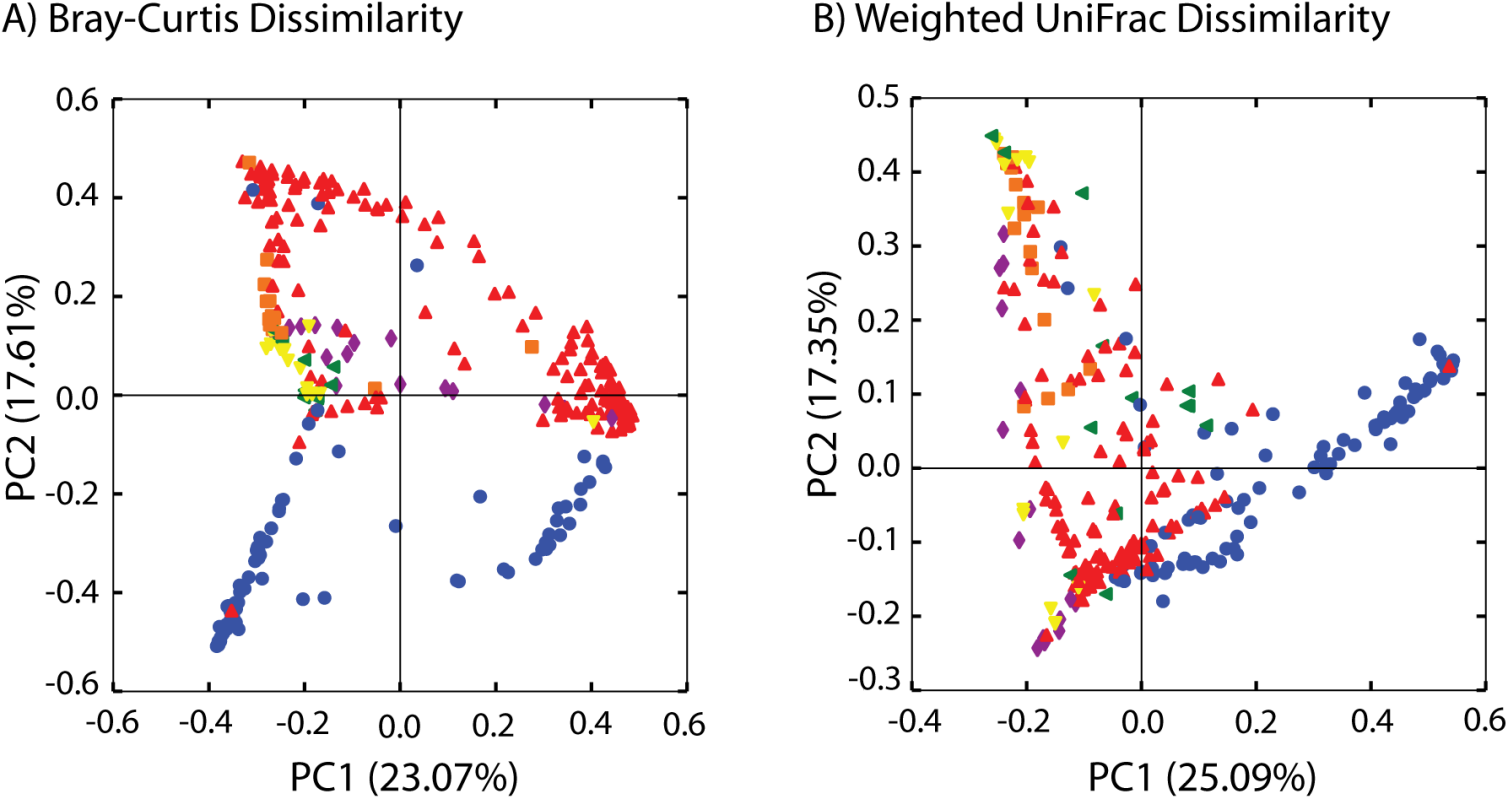
Principal coordinate analysis (PCoA) of flea-associated bacterial communities based on flea species. PCoA was performed based on Bray-Curtis dissimilarities (A) and on weighted UniFrac distances (B). The percentage of variation explained by axes one and two are presented in parentheses. Green: *Ctenophthalmus calceatus cabirus*, Purple: *Dinopsyllus lypusus*, Yellow: *Stivalius torvus*, Blue: *Xenopsylla brasiliensis*, Red: *Xenopsylla cheopis*, Orange: *Xenopsylla nubica*.

The flea species had a large and significant effect on the bacterial community (Fig 3; Table 4); site, rodent host, and elevation (above 1,300m vs. below 1,300m) also varied significantly with community composition when all samples were analyzed simultaneously (Table 4). The effects of host, site, collection date, and sex on bacterial communities within flea species varied depending on the flea species, the factor analyzed, and the metric used to compare communities (Table 5). The effect of sex was widespread across different flea species with males and females having different bacterial communities in *D*. *lypusus* (UniFrac only), *S*. *torvus*, *X*. *brasiliensis*, and *X*. *cheopis*. Host, site, and collection date also significantly affected bacterial communities in *Xenopsylla* species, depending on the metric used to compare communities (Table 5).

**Table 4:** Analysis of Similarity of bacterial communities (All Samples).

	Species		Site		Host		Elevation*	
	R	p-value	R	p-value	R	p-value	R	p-value
**Bray-Curtis**	**0.601**	**0.001**	**0.237**	**0.001**	**0.110**	**0.001**	**0.420**	**0.001**
**UniFrac**	**0.343**	**0.001**	**0.151**	**0.001**	**0.106**	**0.001**	**0.245**	**0.001**
**Weighted UniFrac**	**0.458**	**0.001**	**0.105**	**0.001**	**0.110**	**0.001**	**0.278**	**0.001**

*: Elevation was categorized as above 1300 meters (plague positive) and below 1300 meters (plague negative).

**Table 5:** Analysis of Similarity of bacterial communities (Within Flea Species).

	Host		Site		Date		Sex	
	R	p-value	R	p-value	R	p-value	R	p-value
**Bray-Curtis**								
*C*. *cabirus*	-0.25	0.985	-	-	-	-	-0.17	0.918
*D*. *lypusus*	-0.12	0.799	-0.06	0.632	-	-	0.04	0.253
*S*. *torvus*	-	-	-	-	-	-	**0.75**	**0.001**
*X*. *brasiliensis*	**0.07**	**0.039**	**0.10**	**0.002**	**0.19**	**0.001**	**0.15**	**0.001**
*X*. *cheopis*	**0.05**	**0.021**	**0.12**	**0.001**	0.00	0.388	**0.44**	**0.001**
*X*. *nubica*	0.28	0.182	0.09	0.232	-	-	-	-
**UniFrac**								
*C*. *cabirus*	-0.09	0.742	-	-	-	-	0.02	0.309
*D*. *lypusus*	-0.21	0.911	-0.06	0.618	-	-	**0.16**	**0.038**
*S*. *torvus*	-	-	-	-	-	-	**0.39**	**0.007**
*X*. *brasiliensis*	**0.17**	**0.001**	0.05	0.23	0.09	0.017	**0.08**	**0.003**
*X*. *cheopis*	**0.08**	**0.004**	**0.06**	**0.001**	**0.05**	**0.004**	**0.21**	**0.001**
*X*. *nubica*	0.30	0.086	0.28	0.019	-	-	-	-
**Weighted UniFrac**								
*C*. *cabirus*	-0.11	0.805	-	-	-	-	-0.10	0.818
*D*. *lypusus*	-0.06	0.612	-0.09	0.774	-	-	0.03	0.263
*S*. *torvus*	-	-	-	-	-	-	**0.74**	**0.001**
*X*. *brasiliensis*	**0.11**	**0.002**	**0.11**	**0.002**	**0.20**	**0.001**	**0.06**	**0.012**
*X*. *cheopis*	**0.08**	**0.009**	**0.10**	**0.001**	-0.02	0.821	**0.28**	**0.001**
*X*. *nubica*	0.10	0.209	0.00	0.428	-	-	-	-

Elevation, mean annual precipitation, and average monthly temperature significantly co-varied with bacterial community composition across all samples (Table 6). Within *X*. *cheopis*, bacterial communities co-varied with elevation, mean annual precipitation, and average monthly temperature; within *X*. *brasiliensis*, bacterial communities co-varied with average monthly precipitation (Table 6).

**Table 6:** Mantel Tests comparing bacterial community structure to environmental parameters.

	Elevation		MAP		Precipitation		Temp (avg)	
	R	p-value	R	p-value	R	p-value	R	p-value
**Bray-Curtis**								
All samples	**0.28**	**0.001**	**0.20**	**0.001**	-0.02	0.337	**0.16**	**0.001**
*X*. *brasiliensis*	0.01	0.842	0.05	0.087	**0.14**	**0.001**	**0.08**	**0.021**
*X*. *cheopis*	**0.12**	**0.001**	**0.15**	**0.001**	0.00	0.978	**0.08**	**0.001**
**UniFrac**								
All samples	**0.19**	**0.001**	**0.12**	**0.001**	-0.01	0.712	**0.07**	**0.001**
*X*. *brasiliensis*	-0.01	0.750	0.04	0.273	0.06	0.191	**0.10**	**0.003**
*X*. *cheopis*	0.00	0.965	-0.04	0.104	0.00	0.968	-0.02	0.503
**Weighted UniFrac**								
All samples	**0.17**	**0.001**	**0.11**	**0.001**	-0.04	0.123	**0.11**	**0.001**
*X*. *brasiliensis*	0.01	0.759	0.05	0.096	**0.15**	**0.003**	**0.10**	**0.009**
*X*. *cheopis*	**0.12**	**0.002**	**0.15**	**0.001**	-0.01	0.784	**0.07**	**0.036**

MAP: Mean Annual Precipitation, Precipitation: Average monthly precipitation.

## Discussion

Bacterial lineages within Actinobacteria, Bacteroidetes, Firmicutes, and Proteobacteria dominated community membership of the six flea species examined in this study (Fig 1). These same bacterial phyla have repeatedly been shown to dominate bacterial communities in previous studies of fleas [9,10], other disease vectors [9,32–35], a wide diversity of insects [36–38], and animals in general [39–41]. It is worth nothing that no primer pair will detect all bacteria and that primer choice will always affect detection of bacterial lineages, but the region analyzed here has low non-coverage rates for most phyla (exceptions include Aquificae, Armatimonadetes, Chlamydiae, Planctomycetes, and Verrucomicrobia; these lineages are not common members of insect-associated bacterial communities) [42].

Many of the most common lineages detected are related to known symbionts previously detected in fleas (e.g. *Bartonella* spp. [43–45], *Cardinium* sp. [46], *Wolbachia* spp. [44,47–49], *Spiroplasma* spp. [46,50,51], *Lariskella* sp. [52]). However, some lineages commonly detected in this study are not related to known insect symbionts. Three of the most common lineages were within the Pasteurellaceae family and were abundant within a single flea species but rare or non-existent in other flea species (Table 3). This pattern suggests species-specific symbiosis between the flea species and its corresponding Pasteurellaceae lineage. While this is common with certain groups of bacteria (e.g. Rickettsiales, Bacteroidetes), this species-specific relationship has not been seen previously within the Pasteurellaceae. The Pasteurellaceae lineages discovered here share only 90–95% sequence similarity with other previously sequenced bacteria, suggesting that the lineages discovered here represent new lineages of insect-associated bacterial symbionts.

Flea species had the greatest effect on bacterial community composition (Table 4; Fig 3). This supports previous work that demonstrated a substantial effect of insect host taxonomy on bacterial community composition [36,37]. However, our results differ from previous research on flea-associated bacterial communities that found no differences in bacterial communities between flea species [9,10]. Here, *Wolbachia* spp. and *Bartonella* spp. were commonly found in different flea species, but each flea species also harbored a unique bacterial lineage (Table 3). These lineages unique to specific flea species are likely responsible for the effect of flea species on community composition (Table 4).

To our knowledge, this is the first study to assess environmental effects on insect-associated bacterial communities. We were able to compare flea-associated bacterial communities to environmental variables such as temperature, precipitation, and elevation (Table 6). Elevation, mean annual precipitation, and mean monthly temperature all co-varied significantly with bacterial community composition when using all flea samples (Table 6). This is result is somewhat driven by non-random distribution of flea species across sites (Table 1) and the strong effect of flea species on bacterial community composition (Table 4). Nevertheless, significant effects of elevation, mean annual precipitation, and temperature are also found within *X*. *cheopis*, suggesting that environmental effects may contribute to bacterial community composition. The environmental effects are rather weak, however, and this is somewhat surprising because outbreaks of *Y*. *pestis* are often attributed to environmental change [3,5,53–55].

Bacterial communities of *X*. *brasiliensis* and *X*. *cheopis* changed slightly across the collection periods. A previous study of flea-associated bacteria found communities to vary substantially across time [10], but that study compared bacterial communities collected three years apart whereas this study spans only six months. It is becoming increasingly clear that although insect species harbor unique symbionts within an insect population, the dominant symbionts within a population shift across time and among populations. For example, here a common symbiont of *D*. *lypusus* was a *Lariskella* sp. (Table 3), which has previously been detected in *X*. *cheopis* and a variety of stinkbugs [52]; here it was rarely detected in *X*. *cheopis*, demonstrating both its ability to colonize different insect hosts and its variability of prevalence across populations. This pattern is seen across many insect-associated bacteria and is likely due to a combination of stochastic effects and fitness benefits for the insect of particular insect-bacteria associations. If these symbionts interact with pathogens such as *Y*. *pestis*, their presence or absence may alter the likelihood of successful plague transmission and their variable prevalence across flea-subpopulations may contribute to the patchy distribution in both time and space of plague epizootics.

## Supporting Information

S1 File Mapping file for Dataset. (TXT)

S2 File OTU map. (TXT)

S3 File Representative OTU sequences. (TXT)
